# ADAMTS‐13 and von Willebrand factor: a dynamic duo

**DOI:** 10.1111/jth.13898

**Published:** 2017-12-02

**Authors:** K. South, D. A. Lane

**Affiliations:** ^1^ Centre for Haematology Imperial College London London UK

**Keywords:** ADAMTS‐13, conformational activation, hemostasis, TTP, VWF

## Abstract

von Willebrand factor (VWF) is a key player in hemostasis, acting as a carrier for factor VIII and capturing platelets at sites of vascular damage. To capture platelets, it must undergo conformational changes, both within its A1 domain and at the macromolecular level through A2 domain unfolding. Its size and this function are regulated by the metalloproteinase ADAMTS‐13. Recently, it has been shown that ADAMTS‐13 undergoes a conformational change upon interaction with VWF, and that this enhances its activity towards its substrate. This review summarizes recent work on these conformational transitions, describing how they are controlled. It points to their importance in hemostasis, bleeding disorders, and the developing field of therapeutic application of ADAMTS‐13 as an antithrombotic agent in obstructive microvascular thrombosis and in cardiovascular disease.

## Introduction

**Figure 1:**
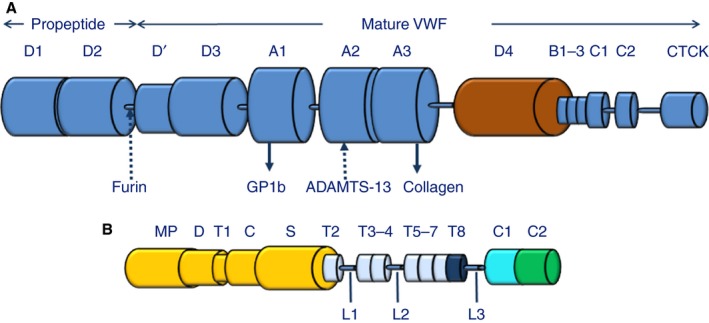
The domain organization of von Willebrand factor (VWF) and ADAMTS‐13. (A) VWF contains multiple functional domains, beginning with domains D1 and D2 at the protein N‐terminus. These domains form the propeptide, and are removed by furin cleavage to generate the mature VWF monomer. The D′ and D3 domains, which are involved in multimer formation, proceed the three central A domains. The A1 domain contains the platelet glycoprotein (GP) 1b‐binding motif, which is exposed under conditions of increased shear stress. Under these conditions, the ADAMTS‐13 cleavage site within the A2 domain is also exposed. The A3 domain contains the constitutively exposed collagen‐binding site responsible for VWF tethering at sites of vascular injury. The D4 domain, shown in brown, binds to the C‐terminal domains of ADAMTS‐13 (see Fig. 3) and initiates ADAMTS‐13 conformational activation. The remaining C‐terminal domains are annotated as they most commonly appear in the literature 108, 109, with the C‐terminal cysteine knot (CTCK) domain, required for dimer formation, at the C‐terminus. (B) ADAMTS‐13 consists of the N‐terminal metalloprotease (MP), disintegrin‐like (D), thrombospondin (TSP) 1 (T1), cysteine‐rich (C) and spacer (S) domains (yellow). The C‐terminal CUB1 (teal) and CUB2 (green) domains are connected to the proximal domains through seven further TSP repeats (TSP2–7 in light blue, and TSP8 in dark blue). Three flexible linker regions (L1, L2, and L3) allow the formation of the ADAMTS‐13 closed conformation (see Fig. 3).

Von Willebrand factor (VWF) is a large and heterogeneous, multidomain adhesive glycoprotein (GP) (Fig. 1A) that is essential for normal hemostatic function. It is synthesized in endothelial cells (and megakaryocytes) as a monomer that dimerizes in the endoplasmic reticulum through a C‐terminal disulfide bond 1. Multimerization occurs in the Golgi, resulting from propeptide‐induced disulfide bond formation 2, 3. Numerous and complex glycan chains are added during synthesis 4, 5. Heterogeneous VWF and ultralarge VWF (ULVWF) are stored within Weibel–Palade bodies, from which they can be released constitutively and upon demand 6. The ULVWF released into the circulation is potentially toxic, because, unprocessed, it is able to spontaneously interact with platelets, forming clumps that can block the microcirculation 7. The pioneering work of Furlan *et al*. and Tsai *et al*. in 1996 identified a VWF‐processing plasma protease with unusual properties, Zn^2+^ and Ca^2+^ dependencies, with an (*in vitro*) requirement for substrate denaturation prior to cleavage 8, 9. The VWF‐cleaving protease is able to reduce the size and hemostatic function of VWF *in vivo* by controlled cleavage at a single, specific site (its scissile bond, Tyr1605‐Met1606) within the VWF A2 domain. The resultant processed VWF circulates as a folded, quiescent and globular protein, which is heterogeneous with respect to its size. Despite this quiescent state, it is able to recognize and bind to collagen exposed by vascular injury through its A3 domain 10, 11. Shear stress unfolding of the protein then reveals the platelet capture site, on the A1 domain, enabling primary hemostasis with the formation of the initial platelet plug 12.

The major motivation for understanding the function of the VWF‐cleaving protease came from the demonstration that its deficiency was the cause of the fatal disease thrombotic thrombocytopenic purpura (TTP) 13, 14, 15 and from the identification of its gene, designated *ADAMTS13* 16, 17, 18, 19, 20 (Fig. 1B). ADAMTS‐13 was recognized as a metalloproteinase family member, with Glu225 at its active site, requiring Zn^2+^ occupancy of three His residues to confer functionality to the proteinase domain 21. Its function also depends on Ca^2+^, and a high‐affinity functional Ca^2+^‐binding site has been identified near the active site 22. In the past 15 years, the role(s) of many of the domains of ADAMTS‐13 have been elucidated. What has also emerged is the pivotal role of the ADAMTS‐13–VWF axis in regulating hemostasis. Severe deficiency of ADAMTS‐13, either congenital or acquired, can result in excess ULVWF and causes TTP. Furthermore, there is increasing evidence suggesting that even a moderate decrease in ADAMTS‐13 activity can also predispose to cardiovascular disease. Regional domain mutation of VWF, on the other hand, increases proteolytic susceptibility to ADAMTS‐13, causing a bleeding disorder, type 2A von Willebrand disease (VWD).

A number of reviews have summarized the progress in understanding the basic mechanisms of ADAMTS‐13–VWF interactions or their role in disease 23, 24, 25, 26, 27, 28, 29, 30, 31, 32, 33. The aims of this review are to provide an update of recent mechanistic work, focusing particularly on the dynamics of interaction, and then to summarize recent experimental and clinical investigations that point to possible therapeutic roles for ADAMTS‐13.

## Shear‐induced conformational unfolding of VWF

The transition of VWF by shear stress from its quiescent, compact folded state to an elongated platelet capture protein is central to its function. Interest has focused on the VWF A2 domain, as this has an obvious structural adaptation allowing response to shear, i.e. its lack of a domain‐spanning disulfide bond. A pioneering single‐molecule pulling and relaxing study of the isolated A2 domain employed double‐stranded DNA handles coupled to beaded tags and optical tweezers, with laser trap detection of force changes induced with a micropipette 34. This demonstrated A2 unfolding at a force of ~ 10 pN. Subsequent studies of incorporation of the A2 domain into an A1–A2–A3 domain construct found doubling of the force required for unfolding 35, 36. Remarkably, force‐induced unfolding completely extends the A2 domain from 1 nm (the distance between the N‐terminus and the C‐terminus) to 58 nm, a similar length to that of the resting VWF monomer. This large extension is accompanied by accelerated cleavage of the domain in the presence of ADAMTS‐13 (see below), arising from exposure of the otherwise hidden exosites that bind ADAMTS‐13 and also exposure of the cleavage site. This sequence of unfolding, extension and cleavage has been elegantly demonstrated by the use of VWF strings under flow 37. As force is relaxed, the domain can spontaneously refold, restricting the action of ADAMTS‐13 34.

**Figure 2:**
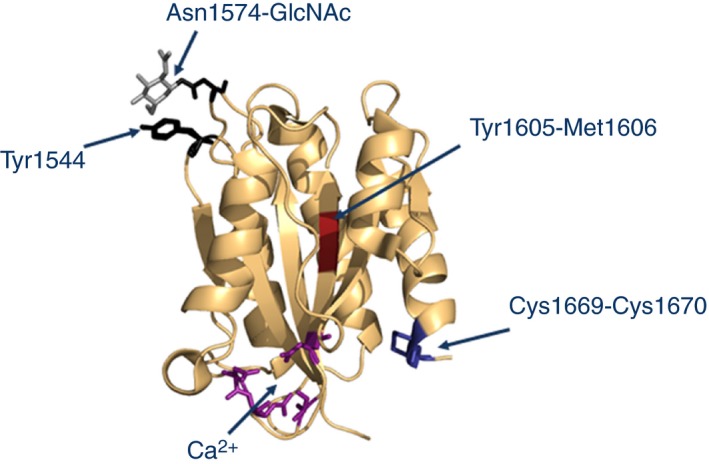
The stabilizing structural features of the von Willebrand factor (VWF) A2 domain. In its folded conformation, the scissile bond (red) of the VWF A2 domain, is cryptic. This conformation is dependent on three specialized structural elements. First, the vicinal disulfide bond (dark blue), at the domain's C‐terminus, forms a hydrophobic plug. Second, the Asp1596, Arg1597, Ala1600 and Asn1602 (purple) form a Ca^2+^ coordination site. Finally, the GlcNAc moiety (gray) of the N‐linked glycan at Asn1574 interacts with Tyr1544 in the adjacent loop (both residues shown in black). This figure was generated in pymol by use of the VWF A2 domain crystal structure (Protein Data Bank 3ZQK 40).

The three principal structural elements controlling A2 domain unfolding and refolding have been identified, and are shown in Fig. 2. An unusual disulfide bond between two vicinal cysteines, Cys1669 and Cys1670, at the C‐terminus of the domain was identified in a crystal structure 38. It was proposed that this bond forms a hydrophobic plug that could improve thermal stability and protect against shear unfolding, inhibiting proteolysis by ADAMTS‐13, a suggestion that was subsequently confirmed 39. An alternative crystal structure identified a Ca^2+^‐binding site formed by Asp1596, Arg1597, Ala1600, and Asn1602 40. Full occupancy of this site in blood is predicted by its micromolar affinity for Ca^2+^. With the use of single‐molecule optical tweezers, force‐induced transitions in unfolding and refolding of the A2 domain were shown to depend on occupancy of this Ca^2+^‐binding site. The third important structure controlling unfolding is the N‐linked glycan at Asn1574. Early studies of the action of ADAMTS‐13 against VWF had demonstrated that occupancy of Asn1574 by its glycan impeded the action of the proteinase 5. It was suggested that this might arise from steric effects of the bulky glycan impeding access of the proteinase to the scissile bond. However, a recent investigation of the role of this glycan has suggested that its inhibitory effect is manifest even when its glycan chain is reduced to its first GlcNAc residue 41. Moreover, the thermal stability of the domain is enhanced by Asn1574‐GlcNAc. It was suggested that Asn1574‐GlcNAc is able to stabilize the domain by interacting with the adjacent loop, containing Tyr1544. With the use of thermal unfolding as a measure of stability, complemented by proteolysis experiments with ADAMTS‐13, it was shown that these three structural features interact in a cooperative manner to provide resistance to unfolding and cleavage 41, 42. As stability is progressively lost, the isolated A2 domain becomes increasingly susceptible to proteolysis, as is also observed in type 2A VWD, owing to A2 domain residue mutation (see also below).

In addition to regulating the susceptibility of VWF to ADAMTS‐13 cleavage, shear‐induced unfolding of VWF has been suggested to play a role in its clearance from plasma. Two studies found that the uptake of VWF by macrophages, through the LDL receptor‐related protein 1 (LRP1) scavenger receptor, was dependent on the presence of shear stress, and therefore concluded that unfolding of VWF is required for recognition by this receptor 43, 44. The work of Chion *et al*. has demonstrated that LRP1 recognition of VWF is dependent on the exposure of a cryptic binding site within the A1 domain, and that the removal of stabilizing glycans from the A2 domain enhances this recognition by accelerating A2 domain unfolding 45. Macrophage LRP1‐mediated clearance of VWF is one of several possible VWF clearance mechanisms, and is the only pathway for which conformation dependence has been demonstrated. This may serve to specifically remove circulating, spontaneously elongated high molecular weight VWF multimers arising from conditions of pathologic shear stress (i.e. stenotic vessels) or ADAMTS‐13 deficiency (TTP), and may prevent the formation of microthrombi under these circumstances.

Unfolding of VWF also depends on domain–domain interactions. This was investigated recently with single‐molecule force measurements performed on VWF dimers 46. When the dimer was stretched from its N‐termini (the D′ and D3 domains), A2 unfolding events were observed at 20 pN, compatible with the isolated A2 force unfolding outcomes described above. However, an additional unfolding event was observed between 50 pN and 120 pN. This was shown to originate from the forced opening of the dimer, not by domain extension, but by dissociation of cation‐dependent binding between D4 domains 46. It is conjectured that initial VWF unfolding arises within the A2 domain, and that the additional length of the molecule is then exposed to a dramatic increase in hydrodynamic force that can subsequently trigger dimer unfolding and full unfolding of VWF 46.

## Conformational activation of ADAMTS‐13 by VWF

The study of ADAMTS‐13 function has been greatly facilitated by the use of truncation mutants, prepared mostly from constructs with progressive deletion from the C‐terminus. The early study of Gao *et al*. revealed that cleavage of the short VWF73 substrate was enhanced approximately four‐fold when full‐length (FL) ADAMTS‐13 was truncated to its MDTCS variant 47. This finding suggested that the distal domains comprising thrombospondin (TSP) repeats and CUB domains might autoinhibit the activity of the molecule, a suggestion that was pursued in two recent studies. Muia *et al*. first used activity assays to determine the effect of pH on ADAMTS‐13 activity 48. They found that FL ADAMTS‐13, but not the truncated MDTCS variant, was most active at pH 6. Furthermore, they observed that mAbs directed at the C‐terminal domains of ADAMTS‐13 could enhance ADAMTS‐13 activity only at pH 6. This suggested an autoinhibitory role for the C‐terminal domains at physiologic pH. In addition to decreased pH and the presence of activating mAbs, this autoinhibition was also shown to be relieved by the presence of the C‐terminal D4 domain of VWF. Interestingly, proteolytic fragments of the VWF D4 domain were unable to activate ADAMTS‐13, suggesting the presence of more than one ADAMTS‐13‐binding site within the D4 domain, an idea that will be returned to below. Using small‐angle X‐ray scattering measurements of ADAMTS‐13 and its truncated variants, Muia *et al*. suggested that ADAMTS‐13 has a folded domain structure, with maximum dimensions that are compact as compared with those suggested by molecular models of extended ADAMTS‐13. This was further supported by electron microscopy (EM), which showed ADAMTS‐13 in both compact and elongated conformations 48.

**Figure 3:**
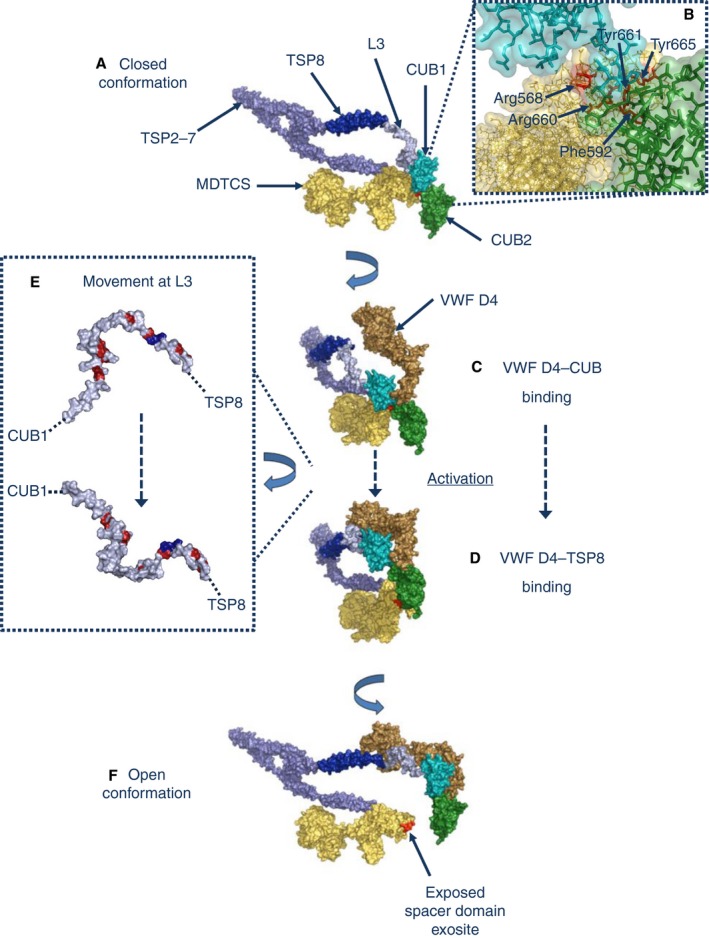
A model for conformational activation of ADAMTS‐13 by von Willebrand factor (VWF). (A) The closed conformation of ADAMTS‐13 results from binding of the N‐terminal MDTCS domains (yellow) to the C‐terminal CUB domains. Proximity of the N‐terminal and C‐terminal regions is enabled by a looped organization of the thrombospondin (TSP) 2–7 (light blue) and TSP8 (dark blue) domains and the three flexible linker regions. (B) Both CUB1 (teal) and CUB2 (green) bind to exosite 3 of the spacer domain (red). The autoinhibitory CUB1 domain is expected to be in close proximity to the key residues Tyr661 and Tyr665. (C) Binding of the D4‐CK domains (predominantly D4, shown in brown) of VWF to both of the CUB domains may function to position D4, and may also destabilize the CUB–spacer interaction. (D) Simultaneous binding of D4 to the TSP8 domain induces the conformational change required for activation. (E) Adoption of the open conformation is thought to be facilitated by the flexible linker region L3. This region is rich in Pro residues (red), resulting in a kinked structure in the closed conformation of ADAMTS‐13 (top). A cluster of Ala residues (purple) impart flexibility and allow the repositioning of the N‐terminal portion of L3 in the open conformation of ADAMTS‐13 (bottom), and hence the movement of the CUB domains. (F) In the resulting open conformation, exosite 3 of the spacer domain is exposed. Adapted from South *et al*. 54. The three‐dimensional model of ADAMTS‐13 was generated in pymol by use of the DTCS crystal structure (Protein Data Bank [PDB] 3GHM 110, homology modeling of the metalloprotease–disintegrin‐like domains, homology modeling of the CUB domains, and the TSP1 repeat crystal structure (PDB 1LSL 111).

South *et al*. approached the same issue by study of a gain‐of‐function (GoF) ADAMTS‐13 spacer domain variant (Tyr568Lys/Phe592Tyr/Arg660Lys/Tyr661Phe/Tyr665Phe, originally described by Jian *et al*. 49), that shows enhanced activity as compared with wild‐type (WT) ADAMTS‐13. Using functional assays in the presence and absence of the ADAMTS‐13 C‐terminal binding fragment of VWF, VWF D4‐CK, they showed that VWF binding to WT ADAMTS‐13 resulted in its unfolding and functional activation 50. However, the GoF variant was found to be in a preactivated state, and could not be further activated by VWF D4‐CK. Direct binding between the spacer domain of the ADAMTS‐13 N‐terminal fragment, MDTCS, and a CUB1–2 domain fragment was also demonstrated. It was proposed that it is this interaction that maintains the closed conformation of ADAMTS‐13 and is disrupted in the GoF variant (Fig. 3A). Interestingly, the GoF variant residue substitutions were engineered at residues that were recognized by autoantibodies causing acquired TTP. It appears that these antigenic determinants are normally masked by CUB binding to the spacer domain, and are only revealed during conformational activation (Fig. 3F). Extensive EM analysis, generating class averages of ~ 6000 particles, confirmed that WT ADAMTS‐13 adopts a compact, globular conformation that is partially relieved in the GoF variant 50.

The activation of ADAMTS‐13 by the VWF D4‐CK fragment highlights the important role of the C‐terminal domains of VWF in its proteolytic processing. Two prior studies had identified the involvement of this region of globular VWF in the recognition of ADAMTS‐13. Using a combination of binding techniques and functional assays, Zanardelli *et al*. had identified an ADAMTS‐13‐binding site in VWF D4‐CK, largely preserved within the D4 domain, that they claimed functioned to approximate the two proteins in the absence of conformational activation of VWF 51. Feys *et al*. used complementary approaches, including immunoprecipitation, to show reversible association of ADAMTS‐13 with globular VWF 52. In both investigations, the binding interaction was estimated to be of modest affinity, i.e. ~ 80 nm, and a stoichiometry of approximately one ADAMTS‐13 molecule to 250 VWF molecules was suggested 51, 52. At the time of these reports, it was believed that this binding was primarily for positioning of the two, in readiness for shear‐induced VWF unfolding, in order for cleavage to occur efficiently. In the light of the above recent work demonstrating conformational activation of ADAMTS‐13, the binding reaction can also be viewed as inducing an activation step to prepare ADAMTS‐13 for its exosite interactions with the shear‐induced unfolded VWF A2 domain (see also below). In the circulation, only 3% of ADAMTS‐13 is thought to be bound to globular VWF 52, presumably in a conformationally active state. Under thromboinflammatory conditions, during which VWF is released from Weibel–Palade bodies of the activated endothelium, or is tethered to the exposed subendothelium, the proportion of conformationally active ADAMTS‐13 may be much higher.

The demonstration of conformational activation of ADAMTS‐13 prompted an investigation by Deforche *et al*. of the flexibility of the distal ADAMTS‐13 domains 53. They generated a series of mAbs with differing specificities. One of these recognized a normally hidden epitope in the proximal domains that was exposed by activating anti‐ADAMTS‐13 antibodies. This was used to probe the role of three linker regions within the distal TSP2–CUB2 domains that contributed to the structural flexibility needed to accommodate the changes from compact to more elongated shapes during the activation process. Deletion of these linker regions resulted in increased recognition of the cryptic epitope, indicative of an open conformation and therefore enhanced activity of ADAMTS‐13.

The work of South *et al*. has refined the model of ADAMTS‐13 conformational activation and the mechanism by which the autoinhibitory CUB–spacer domain interaction is interrupted by binding of the VWF D4‐CK domains 54. Using direct binding analysis (surface plasmon resonance [SPR]), they first determined that both the CUB1 domain and the CUB2 domain bind to exosite 3 of the spacer domain. Both of these interactions are abolished in the GoF variant. The orientation of the CUB domains is such as to allow both CUB1 and CUB2 to interface with Arg568, Phe592, Arg660, Tyr661, and Tyr665 (Fig. 3B). Only the isolated CUB1 domain has an inhibitory effect in activity assays of MDTCS, which is in agreement with this model, in which it is positioned in close proximity to the key residues Tyr661 and Tyr665. South *et al*. next addressed the question of how VWF D4‐CK binding disrupts the closed conformation of ADAMTS‐13 54. It was determined, with SPR, that a VWF D4‐CK domain fragment binds with moderate affinity to both CUB1 and CUB2 (Fig. 3C). This suggested that binding of the VWF C‐terminal domains may compete with the CUB–spacer domain interaction. However, removal of the CUB domains did not abolish binding of VWF D4‐CK to ADAMTS‐13. An explanation for this was provided when they showed that TSP8 is a major binding partner of VWF D4‐CK, and that this binding interaction is essential for conformational activation (Fig. 3D). This new model of activation, with binding of VWF D4‐CK to TSP8, CUB1, and CUB2, confirms the functional importance of the flexible linker region L3 described by Deforche *et al*. (Fig. 3E). This new model also supports the idea, suggested by Muia *et al*., that VWF D4 contains multiple binding motifs for ADAMTS‐13 48.

The transition from compact to elongated structures has brought to the fore the intriguing issue of the restricted specificity of ADAMTS‐13. It is interesting that this metalloproteinase has only a single known function, that of cleavage of VWF. Other than suggested inhibition of ADAMTS‐13 by neutrophil peptides 55, there are no known physiologic inhibitors to regulate its action. It is rare that proteases are completely specific for a single substrate, and local factors, such as regional concentrations of protease and substrate, can determine whether there is sufficient availability to allow proteolysis to occur. Promiscuity may therefore be expected. The entire range of possible residue changes that permit proteolysis of the small VWF substrate VWF73 has been mapped by use of a substrate phage display library 56, 57. This has highlighted the importance of the scissile bond residues as well as previously identified exosite regions (see below), but has also demonstrated that, within these regions, many possible residue substitutions that permit cleavage are possible. This suggests that sequence similarities found within other proteins might encourage their cleavage. To explore this, South *et al*. recently investigated whether the compact structure of ADAMTS‐13 might restrict its substrate specificity. In the course of flow experiments, in which VWF/fibrin(ogen)‐containing pseudothrombi were lysed by ADAMTS‐13, it was found that fibrinogen could also be cleaved by the protease, under circumstances in which the protective spacer–CUB interaction was prevented or disrupted 58. They have shown that unfolded, activated ADAMTS‐13 (GoF ADAMTS‐13, the truncated variant MDTCS and WT ADAMTS‐13 activated by VWF D4‐CK) can directly cleave the Aα chain of fibrinogen, rendering fibrin formed in thrombi more prone to lysis by the fibrinolytic system. The sequence surrounding the cleavage site in the Aα chain resembled, but was not identical to, the cleavage site in VWF. It was therefore suggested that conformational quiescence of ADAMTS‐13 may help to protect against off‐target proteolysis.

This study has generated some debate regarding the secondary proteolytic potential of ADAMTS‐13. In a letter to the *Journal of Thrombosis and Haemostasis*, Cao and Zheng suggested that proteolysis of fibrinogen by ADAMTS‐13 is indirect, and occurs because of the generation of plasmin 59. However, as described by South *et al*. in their reply, the proteolysis observed in the original study occurred at a single site of the Aα chain, and was not sensitive to either PPACK or tranexamic acid 60. Therefore, in these experiments, extensive plasminogen activation had not occurred. In work carried out subsequent to the original study, Cao *et al*. proposed that conformationally active ADAMTS‐13 can indeed proteolyze plasminogen, generating functional plasmin, similarly to tissue‐type plasminogen activator (t‐PA) 61. It is therefore possible that conformationally active ADAMTS‐13 may have more than one secondary substrate, and that it may play a dual role in the fibrinolytic system. It could act directly on fibrinogen, removing the α_2_‐antiplasmin‐binding site and rendering fibrin more susceptible to the action of plasmin, while simultaneously upregulating the generation of plasmin through its proteolytic activity against plasminogen. Unlike the secondary activity of plasmin against VWF, which has been suggested to be a natural backup during periods of ADAMTS‐13 deficiency 62, the physiologic relevance of ADAMTS‐13 secondary activity is yet to be established. Further work is required to confirm some of these suggestions, which may be an important consideration in the development of ADAMTS‐13 variants as therapeutic agents (see below).

Preactivated ADAMTS‐13 variants may have disadvantages in the therapeutic context. If their improved recognition by TTP autoantibodies 50 is not diminished by prior mutation, there may be increased clearance/inhibition by TTP autoantibodies, particularly when they are administered to TTP patients with persisting, circulating inhibitory antibodies 63. The finding that 5% of healthy (non‐TTP) individuals are positive for circulating low‐affinity anti‐ADAMTS‐13 IgG autoantibodies 64 may suggest that administration of preactivated ADAMTS‐13 in other indications should be approached cautiously. Nevertheless, unlike for VWF, there is no evidence that the ADAMTS‐13 conformation affects its cellular, non‐autoantibody‐mediated clearance. Although there is an obvious glycan‐dependent difference in the half‐life of ADAMTS‐13 (expressed in different cell lines) when it is injected into mice 65, 66, there is no observable difference between the half‐lives of FL ADAMTS‐13 and its truncation variants T8 and S (which are presumably conformationally active) when they are used in animal models 66. In individuals with no circulating ADAMTS‐13 autoantibodies, it is therefore likely that the therapeutic administration of preactivated ADAMTS‐13 would result in enhanced cleavage of ULVWF.

## Exosite interactions leading to cleavage of the VWF scissile bond

The force‐induced unfolding of the VWF A2 domain reveals cryptic exosites that progressively increase the affinity of binding between ADAMTS‐13 and VWF. The earliest characterized of these was that contained within the VWF sequence, i.e. Glu1660–Arg1668 67, 68. This exosite interacts with the spacer domain of ADAMTS‐13, i.e. Arg568, Arg660, Tyr661, and Tyr665, which is the antigenic region recognized by autoantibodies generated and found in patients with acquired TTP 69, 70 (see also the GoF ADAMTS‐13 variant mentioned above). An important additional exosite was then located within the unfolded VWF A2 domain by De Groot *et al*., who demonstrated an interaction of Asp1614 of VWF with ADAMTS‐13 disintegrin domain residue Arg349 71.

The deletion mutagenesis approaches used by Gao *et al*. had suggested that the Cys‐rich domain of ADAMTS‐13 might enhance its interaction with unfolded VWF 47, but the mechanism of this was not fully defined until recently. De Groot *et al*. used a targeted glycan incorporation approach to narrow the region on the Cys‐rich domain that might be involved 72. By substituting residues close to a glycan‐modified residue at position 476 that inhibited activity and binding to VWF, they identified a hydrophobic pocket containing Gly471–Val474 that interacts with an exosite in the unfolded VWF A2 domain. This exosite is composed of the hydrophobic residues Ile1642, Trp1644, Ile1649, Leu1650, and Ile1651. Disruption of the exosite by substitution with hydrophilic residues reduced the catalytic efficiency of cleavage of VWF substrates ~ 12‐fold 72.

As well as increasing the affinity of the interaction between ADAMTS‐13 and VWF, these exosites help to bring the active site of the metalloproteinase domain into proximity with the VWF scissile bond. For proteolysis to occur, P1 Tyr1605 and P1′ Met1606 of the VWF substrates must engage with the S1 and S1′ subsites on the face of the active site of the protease. This engagement requires a hydrophobic interaction between VWF P3 Leu1603 and hydrophobic ADAMTS‐13 residues, possibly Leu198, Leu232, and Leu274, collectively forming the S3 subsite 73.

## The potential clinical importance of conformational dynamics

### Bleeding disorders, type 2A VWD, and acquired von Willebrand syndrome

The importance of the dynamic control of ADAMTS‐13 and VWF is well illustrated in patients with the bleeding disorder type 2A VWD, who carry mutations causing residue substitutions within the VWF A2 domain. Numerous disease‐causing mutations are located within the 175 residues of the domain. Such mutations can result in impaired secretion of VWF 74 (termed type 2A group 1) or increase the susceptibility of VWF to proteolysis by ADAMTS‐13 75 (type 2A group 2). Mutations also cause both phenotypes 76. These phenotypes arise directly as a consequence of the conformational flexibility of the A2 domain conferred by the absence of a domain‐spanning disulfide bond: without the rigidity provided by the domain‐spanning disulfide bond, the A‐domain fold is readily disrupted, causing either increased cellular retention or cleavage 77. Although the three stabilizing features described above, i.e. the vicinal cysteines, Ca^2+^‐binding site, and N‐linked glycan at Asn1574, provide some resistance to spontaneous unfolding, the A2 domain appears to be unable to retain its natural fold under the additional pressure of residue substitutions. Because many of these mutations disrupt the stability of the A2 domain conferred by the three structural elements, allowing unfolding and exposure of the exosite binding sites for ADAMTS‐13, they thereby facilitate proteolysis of the scissile bond. Mutations occur throughout the domain and, regardless of their position or nature, there can be enhanced proteolysis, depleting ULVWF multimers and predisposing to or causing bleeding 78, 79.

A second example of disruption of the normal interaction of VWF and ADAMTS‐13 is provided by acquired von Willebrand syndrome associated with a left ventricular assist device (LVAD), recently reviewed by Nascimbene *et al*. 80. LVADs were introduced for use in patients awaiting heart transplantation, to provide temporary artificial circulatory improvement. Shear forces within these devices or their connecting tubes may exceed that of the normal circulatory system. VWF may be abnormally stretched or induced to bind to the surface of the biomaterials components and then to unfold. Excessive unfolding would be expected to be accompanied by increased proteolytic action by ADAMTS‐13. Although direct evidence of enhanced ADAMTS‐13 activity is currently lacking, it is evident that there is a reduction in the level of ULVWF multimers in patients with LVADs 80. This reduction in the level of multimers could contribute to the complex hemostatic problems (bleeding, in particular) that may be experienced by users of LVADs.

### TTP

The activating conformational change in ADAMTS‐13 induced by VWF can be predicted to be important in TTP. Both congenital and acquired TTP are characterized by low levels of circulating ADAMTS‐13 and the subsequent formation of VWF/platelet‐rich microthrombi in the microvasculature. The acquired form of the disease results from the formation of autoantibodies against ADAMTS‐13, which can be inhibitory but can also decrease ADAMTS‐13 antigen levels through increased clearance from the circulation 81. Many of the most common autoantibody epitopes are found within the crucial spacer domain exosites of ADAMTS‐13. South *et al*. demonstrated that one of these epitopes, comprising Arg568, Phe592, Arg660, Tyr661 and Tyr665 in exosite 3 of the spacer domain, was recognized more readily by the patient‐derived mAb II‐1 (first described by Pos *et al*. 82) upon conformational activation of ADAMTS‐13 50. Masking of cryptic epitopes in the closed conformation of ADAMTS‐13 may prevent autoantibody formation. A compact structure of plasma ADAMTS‐13 may explain the low immunogenicity of ADAMTS‐13 administered as replacement with fresh frozen plasma to TTP patients with congenital deficiency.

For autoantibody development, cryptic epitopes may become exposed. Increased levels of plasma VWF during pregnancy or infection, which are common clinical triggers of acquired TTP, might lead to substrate‐induced activation of ADAMTS‐13 and antigenic exposure. Recently, Verbij *et al*. found that CD4^+^ T cells from acquired TTP patients were reactive to CUB2 domain‐derived peptides 83, the sequences of which are found on the exposed surface of CUB2 in the current model of the activated ADAMTS‐13 structure (Fig. 3). The presence of antibodies against the constitutively exposed C‐terminus of ADAMTS‐13 could be the result of epitope spreading, subsequent to initial immune recognition of newly exposed cryptic epitopes. In another study, in which a panel of mAbs against ADAMTS‐13 were generated, numerous antibodies, targeted at the C‐terminal domains of ADAMTS‐13, were identified that enhance its activity to a level that indicates conformational activation 53. Therefore, early antigenic recognition of ADAMTS‐13 may occur through surface‐exposed epitopes within the C‐terminal domains. The potential activating effect of these antibodies may lead to further exposure of the N‐terminal domain epitopes and the development of an inhibitory autoantibody population. This may be compatible with the development of anti‐ADAMTS‐13 IgG in healthy individuals 64. The study by Grillberger *et al*. 64 suggested that low‐affinity, non‐inhibitory antibodies, some of which were targeted to the C‐terminal domains of ADAMTS‐13, may precede the memory B‐cell hypermutation needed for the generation of high‐affinity (and possibly activating) antibodies. It will be interesting to determine whether the normally compact structure of ADAMTS‐13 is perturbed during acute episodes of acquired TTP.

In mouse models of congenital TTP, in which *ADAMTS13* ^−/−^ mice are challenged with recombinant VWF 84, the administration of recombinant ADAMTS‐13 (rADAMTS‐13) ameliorates TTP. Animal models of antibody‐mediated acquired TTP have been developed by inducing ADAMTS‐13 deficiency in mice, rats and baboons by the use of inhibitory mAbs 63, 85, 86. In these models, the clinical features of TTP manifest because of an increase in the circulating ULVWF level. Agents that target ULVWF may therefore have prophylactic value. This has been shown in mice by the use of streptokinase‐induced plasmin generation and in baboons by the use of *N*‐acetylcysteine, both of which reduce ULVWF multimer levels 62, 87. In a rat model of TTP, the administration of rADAMTS‐13 was able to overcome the presence of the inhibitory autoantibodies and degrade ULVWF multimers 63. These animal studies show a clear protective effect of rADAMTS‐13 therapy in both genetically determined and acquired TTP. Two other therapeutic agents, caplacizumab and the aptamer ARC1779, which target the collagen–VWF–GP1b axis, have shown promise in phase II clinical trials of acquired TTP 88, 89. Collectively, these studies argue for the potential of targeting VWF with rADAMTS‐13 as a treatment for TTP. This has recently been supported by a phase I clinical trial in congenital TTP patients, in which rADAMTS‐13 effectively reduced the size of circulating VWF multimers 90.

### VWF–ADAMTS‐13 dynamics in cardiovascular disease

The dynamics of both VWF and ADAMTS‐13 are of potential importance in cardiovascular diseases and their management. It has been suggested that imbalance of their plasma levels (low ADAMTS‐13 and high VWF) might predispose to cardiovascular disease, particularly myocardial infarction (MI) and ischemic stroke (IS) 91, 92. A number of case–control, prospective and meta‐analysis studies have supported this suggestion 93, 94, 95, 96, 97, 98. A study by Bongers *et al*. also established a strong association between ADAMTS‐13/VWF levels and both coronary heart disease and peripheral arterial disease 99. A possible pathogenic role of VWF/ADAMTS‐13 in atherosclerosis has also been suggested. A strong association was identified between high levels of VWF and the progression of atherosclerosis in IS patients 100, and, more recently, VWF was suggested as a predictor of coronary plaque burden and cardiovascular outcome in patients with acute coronary syndrome and stable angina pectoris 101. Whether a low ADAMTS‐13 level might be a risk factor for atherosclerotic plaque progression 102 requires further study.

The above studies of cardiovascular disease were largely conducted by measurement of antigen or simple function assays of ADAMTS‐13 levels, as it is impractical to measure ULVWF levels and the conformational state of ADAMTS‐13 in large studies. To establish whether the moderate changes in VWF and ADAMTS‐13 levels, which appear to be risk factors for IS and MI, are further influenced by the dynamic interplay between the two will require the development of new analytic approaches. Further insights into the roles of the ADAMTS‐13–VWF axis in cardiovascular diseases, and the potential of rADAMTS‐13 as a therapeutic agent, have been obtained with experimental models, some of which are summarized below.

### Animal models of IS and MI

The pivotal work performed by Zhao *et al*. 65, 103 and Gandhi *et al*. 65, 103 established the importance of VWF and ADAMTS‐13 in the pathophysiology of IS and MI by using *VWF* ^−/−^ and *ADAMTS13* ^−/−^ mice. Using a transient middle cerebral artery occlusion (tMCAO) model of cerebral ischemia, Zhao *et al*. demonstrated that VWF deficiency, in both *VWF* ^+/−^ and *VWF* ^−/−^ mice, resulted in a reduction in infarct volume 65. Conversely, ADAMTS‐13 deficiency caused an increase in infarct volume. This was directly attributed to the loss of its action on VWF, as the infarct volumes in *VWF*/*ADAMTS13* ^−/−^ mice were similar to those observed in *VWF* ^−/−^ mice. These findings were mirrored in the study performed by Gandhi *et al*. 103, in which ADAMTS‐13 deficiency was found to increase infarct size, and exacerbate the resulting functional deficit, following acute myocardial ischemia/reperfusion (I/R) injury in mice 103. Again, this was attributed to the action of ADAMTS‐13 on VWF, as infarct size was reduced to a similar extent in both *VWF* ^−/−^ and *VWF*/*ADAMTS13* ^−/−^ mice.

Importantly, in both investigations, the detrimental effect of ADAMTS‐13 deficiency was relieved by targeting VWF, either with neutralizing mAbs 103, or by the administration of rADAMTS‐13 65. The administration of rADAMTS‐13 to WT mice undergoing tMCAO did not induce cerebral hemorrhage and did not sensitize mice in the thrombocytopenia‐induced intracerebral hemorrhage model 65. The precise mechanism by which rADAMTS‐13 protects against ischemic injury remains uncertain. The finding that rADAMTS‐13 administration does not increase bleeding time to the same extent as VWF deficiency 65 suggests that reduction of ULVWF levels, rather than VWF antigen depletion, may be responsible. It is likely that rADAMTS‐13 can lyse existing thrombi and prevent the formation of further thrombi by decreasing the level of circulating ULVWF. The protective role of rADAMTS‐13 in myocardial I/R injury was also demonstrated in the study by De Meyer *et al*. 104. In this study, the detrimental effect of ADAMTS‐13 deficiency on infarct size in a mouse model of acute myocardial I/R injury was demonstrated. Furthermore, it was shown that rADAMTS‐13 administration to WT mice resulted in reduced infarct size, reduced myocardial apoptosis, and reduced neutrophil infiltration into the damaged myocardium 104. Again, no potential hemorrhagic risk or bleeding tendency was observed in animals treated with rADAMTS‐13.

The therapeutic potential of rADAMTS‐13 for the treatment of IS has been investigated 105. It has been suggested to be able to circumvent two of the major limitations associated with t‐PA. First, the administration of rADAMTS‐13 did not induce the blood–brain barrier destabilization and hemorrhagic risk normally associated with t‐PA 105. Second, rADAMTS‐13 appears to have a longer therapeutic time window than t‐PA, as reduced infarct size was observed in animals treated with ADAMTS‐13 when it was administered up to 4 h after injury 106. Denorme *et al*. have examined the composition of thrombi recovered from patients suffering acute cerebral ischemia 107. They found that these clots varied in composition, with the VWF content varying from 6% to 49%. This may account for instances of t‐PA‐refractory occlusions in patients with stroke. Using the focal ischemia mouse model, in which middle cerebral artery occlusion is largely VWF‐dependent, they confirmed that VWF‐rich thrombi are t‐PA‐resistant. They were able to demonstrate a significant, and dose‐dependent, reduction in infarct size and increased recanalization upon administration of rADAMTS‐13. This thrombolytic effect was also partially maintained when ADAMTS‐13 administration was delayed by 1 h.

### Summary

Growing clinical and experimental evidence suggests the importance of the VWF–ADAMTS‐13 axis not only in hemostasis, bleeding disorders, and TTP, but also in cardiovascular disease. The improved knowledge of the dynamics of interactions may first impact on the understanding of these complex diseases, and then also offer the potential for improved interventions.

## Addendum

K. South and D. A. Lane designed, wrote and reviewed the article.

## Disclosure of Conflict of Interests

The authors state that they have no conflict of interest.
